# Atraumatic Splenic Rupture: A Notable Complication of Rivaroxaban Use

**DOI:** 10.7759/cureus.38992

**Published:** 2023-05-14

**Authors:** Catherine A Ostos Perez, Kristina Menchaca, Can X Jones, Erika Ostos Perez, Shaun Isaac

**Affiliations:** 1 Internal Medicine, University of Miami John F. Kennedy Medical Center, Atlantis, USA; 2 Biological Sciences, St. Petersburg College, Clearwater, USA

**Keywords:** medication side-effects, secondary stroke prevention, non-vitamin k oral anticoagulant, newer oral anticoagulants, direct oral anticoagulants (doac), atrial fib, therapeutic anticoagulation, major bleeding events, spontaneous splenic rupture, atraumatic splenic rupture

## Abstract

Direct oral anticoagulants (DOACs) are well known to be associated with bleeding complications. However, little is known about their association with atraumatic splenic rupture, a potentially fatal condition. We present the case of a 73-year-old female with paroxysmal atrial fibrillation managed with rivaroxaban who developed a spontaneous atraumatic splenic rupture.

This highlights the importance of recognizing this complication in patients without previous risk factors, such as abdominal trauma or infiltrative splenic disease, who are under anticoagulation with DOACs. There is a strong need for further research on this complication's underlying mechanism and management.

## Introduction

Direct oral anticoagulants (DOACs), specifically factor Xa inhibitors such as rivaroxaban and apixaban, have drawn lots of attention, considering that it has been demonstrated that they are as effective as Vitamin K antagonists (VKAs) as a treatment for the prevention of ischemic stroke and systemic thromboembolism in patients with atrial fibrillation and venous thromboembolism [1,2]. They also proved to have a better safety outcome, especially regarding the risk of major bleeding [1,2]. They have been non-inferior or even have shown superior efficacy with similar or excellent safety compared with the standard of care at that point (warfarin with or without low-molecular-weight heparin LMWH) in multiple clinical trials [1-3].

Although VKAs and DOACs are associated with a reduction in the risk of stroke in atrial fibrillation patients, they have many complications that result in adverse outcomes in clinical practice, including hemorrhage [1]. The reported annual rates of major bleeding with rivaroxaban or apixaban in the phase III trials for atrial fibrillation were 3.6% and 1.4%, respectively. The corresponding rates in the phase III VTE trials during the average six-month treatment period with rivaroxaban or apixaban were 1.1% and 0.6%, respectively [3]. Several clinical studies have contributed to the inquiry into the hemorrhage outcomes of rivaroxaban versus warfarin.

Rivaroxaban was the first oral factor Xa inhibitor used in clinical practice and provided potential advantages over VKAs, including rapid onset, an offset of action, and fewer drug interactions [1]. However, there is limited data regarding the events of bleeding. According to the International Society on Thrombosis and Hemostasis, major bleeding in non-surgical patients is defined as the ones that are fatal and/or symptomatic in a critical area (intracranial, intraocular, retroperitoneal, intra-articular or pericardial, or intramuscular causing compartment syndrome) and/or bleeding causing a fall of hemoglobin leading to transfusion of two or more units of blood or red blood cells [4].

In a study by Beyer-Westendorf et al. of rivaroxaban-related bleeding events, 6% were major and managed conservatively in 60% of cases, and the rest required surgical treatment [5]. Splenic rupture can be considered major bleeding according to its clinical presentation.

Trauma is the most common cause of a ruptured spleen. The majority of atraumatic spleen ruptures (ASR) develop from a diseased spleen or from disorders that include infections, coagulopathy, and neoplasms [6]. Non-traumatic rupture is rare, but when it does happen, it has an approximate 12% mortality [7].

Due to its prevalence, malaria represents the single major cause of ASR worldwide. However, in the Western world, where there is less prevalence, other etiologies should be assessed. In a study by Kocael et al., it was shown that the most common cause of spontaneous splenic rupture in a non-diseased spleen was the use of anticoagulants [7]. Drug-related ASR cases account for up to one-third (9%-33%). In the anticoagulated population, splenic bleeding is a rare complication compared to the common hemorrhages involving the anterior abdominal wall and iliopsoas muscles [6].

There have only been 12 reported cases of splenic rupture related to direct oral anticoagulants [8-11], and only six of them are due to rivaroxaban [12-16]. We present an unusual case of spontaneous splenic laceration secondary to anticoagulation with rivaroxaban.

## Case presentation

A 73-year-old female came to the Emergency Department with a chief complaint of left-sided, pleuritic-type chest pain and left upper and lower quadrant abdominal pain, which started after dinner. Medical history was significant for hypertension (HTN), heart failure with reduced ejection fraction (HFrEF) with an automated implantable cardioverter defibrillator (AICD), and paroxysmal atrial fibrillation (PAF) (CHA2DS2-VASc Score of 3 points, HAS-BLED Score of 1) managed with rivaroxaban. She did not have any prior history of bleeding, stroke, or complications of anticoagulation. 

She denied any previous surgeries, physical trauma, or allergies. Upon arrival at the ED, vital signs were stable. The physical exam was significant for bilateral rales, trace ankle edema, and 7 cmH2O above the left sternal angle of jugular venous distention. The abdomen was soft and slightly tender in the left upper quadrant without associated guarding or rebound.

Laboratory results showed potassium of 3.4 mEq/L, creatinine of 1.44 mg/dL (eGFR 35.7 mL/min/1.73 m2), beta-natriuretic-peptide (BNP) of 593 pg/ml, hemoglobin of 9.1 g/dL, platelets of 123,000 x103 per microliter, and negative troponin I (<0.01). The electrocardiogram revealed a first-degree atrial-ventricular block, and a chest X-ray showed bilateral lower lobe atelectasis. At that time, the patient received management for decompensated heart failure with IV lasix 80mg IV daily, and anticoagulation was continued with rivaroxaban 15mg daily.

Several hours later, the left upper quadrant abdominal pain worsened and was associated with diaphoresis, dizziness, and nausea. The patient denied other constitutional symptoms, including shortness of breath, vomiting, and genitourinary symptoms. The blood pressure dropped to 70/43 mmHg, which prompted resuscitation with fluids; repeated hemoglobin decreased sharply from 9.1 g/dL to 7.9 g/dL. The digital rectal exam showed no blood.

**Figure 1:**
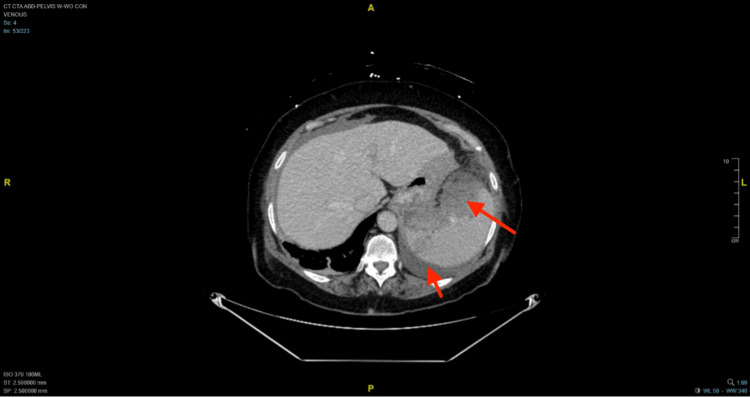
The transverse plane of computerized tomography shows in red arrows the areas of bleeding

**Figure 2:**
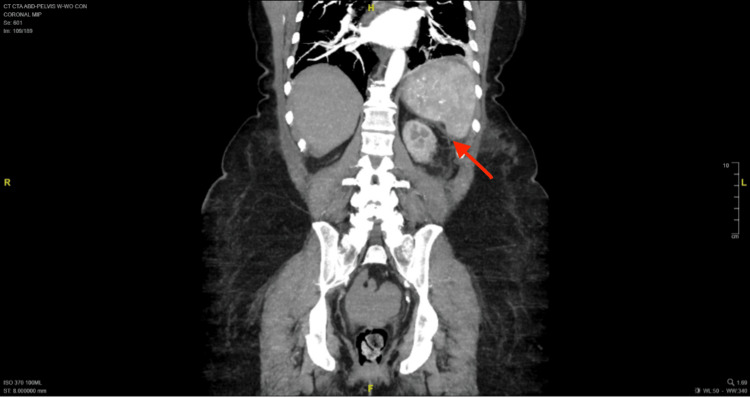
The coronal plane of computerized tomography shows active bleeding as described in the text

Computerized tomography (CT) of the abdomen and pelvis (Figures 1, 2) revealed a moderate amount of hyperdense ascites in the pelvis and a small amount of hyperdense ascites in the abdomen. The density was consistent with a hemorrhagic component, suggesting a splenic origin for intrabdominal bleeding. A CT with an angiogram of the abdomen and pelvis demonstrated a large subcapsular splenic hematoma. The medial aspect of the spleen was noticed to have a punctuate focus of active bleeding. 

Given the acuity of the bleeding, splenic artery embolization was performed with success by interventional radiology, which stopped the bleeding. Anticoagulation was held, and the patient received treatment with a transfusion of two packed red blood cells, with subsequent serial measurements of hemoglobin and hematocrit and imaging until it was determined that there was no more active bleeding. Upon resolution of the episode, the patient was discharged home. The patient’s rivaroxaban was stopped upon discharge, and she was instructed to have outpatient management with follow-up appointments with cardiology and hematology-oncology to determine the appropriate timing to restart anticoagulation.

## Discussion

Atraumatic splenic laceration or rupture is a rare event. It is usually associated with medical conditions leading to infiltration of the spleen, such as infectious mononucleosis, leukemias or lymphomas, amyloidosis, polyarteritis nodosa, or certain medications [17-19]. Orloff and Peskin defined criteria for spontaneous rupture of the normal spleen, which include no history of trauma, no evidence of disease affecting the spleen, no evidence of peri-splenic adhesions or spleen scarring, a normal spleen in gross/histological examination, and no significant rise in viral antibody titers [20]. In our case, the patient denied previous abdominal trauma or any history or current clinical signs of the above-mentioned medical conditions.

There are scattered data on the association between reported DOACs use and atraumatic splenic rupture. The underlying mechanism remains unknown, although some studies suggest that Xa inhibitors may increase bleeding risk by exacerbating undetected splenic microtraumas [8,10]. The concomitant use of medications such as antiplatelets, p-glycoprotein, and CYP 3A4-inhibiting drugs and poor renal function can increase the risk of bleeding with DOACs [15]. In our case, the patient's eGFR of 35 could have been a contributing factor. Any type of splenic rupture is potentially fatal, and we should consider these interactions when managing patients. 

Even though there is no report of the incidence of cases, the occurrence of splenic rupture among patients with DOACs is a rare but significant complication whose pathophysiology is unknown. Although there are risk factors for bleeding, including concomitant use of antiplatelets and NSAIDs [20], uncontrolled hypertension, heavy alcohol use, age, heart failure, and vascular disease at baseline, there is no documented way to predict the risks for splenic rupture.

One of the diagnostic challenges is recognizing splenic rupture in patients without previously reported abdominal trauma or infiltrative disease of the spleen. A high degree of clinical suspicion can make a difference in saving a patient's life. However, left-sided abdominal pain, hypotension, and a decrease in hemoglobin levels should prompt bedside abdominal ultrasound. In our case, no previous risks were reported by the patient that would suggest splenic rupture, except for rivaroxaban use.

Splenic rupture is usually treated surgically, and splenectomy is done for patients with underlying malignancies. However, there is an increasing trend towards non-operative management for ASR [6]. The management of splenic rupture secondary to rivaroxaban depends on the hemodynamic stability of the patient. The initial approach is to stop anticoagulation, volume resuscitation, and, if available, reverse anticoagulation [8]. The current strategy is to provide circulatory support by administering blood products. Guidelines support the administration of prothrombin complex concentrates (PCCs) for managing major bleeding events (MBEs) in patients on rivaroxaban and apixaban. Studies report that most patients with MBE associated with rivaroxaban or apixaban treated with PCCs achieve effective bleeding control with few observed thromboembolic events [3].

Additionally, surgical options such as transcatheter arterial embolization (TAE) and open surgical exploration for hemodynamically unstable patients are available. TAE is a valuable option in cases of splenic rupture, particularly in cases associated with anticoagulation, malaria, and mononucleosis, and when CT detects active arterial bleeding [3]. In our patient, the presence of ongoing bleeding led to TAE.

A study by Lip et al. [19] demonstrates that initiation with apixaban was associated with a significantly lower risk of major bleeding as compared with initiation on warfarin among newly anticoagulated non-valvular atrial fibrillation patients. Furthermore, patients on rivaroxaban or warfarin had a significantly greater risk of major bleeding compared with those initiating apixaban. There was no significant difference in the risk of major bleeding among patients newly initiated on dabigatran compared with apixaban or warfarin initiators [19]. A study by Wayne et al. described an increased risk of bleeding in patients treated with rivaroxaban rather than apixaban; however, more studies are needed to investigate more [20].

Although rare, spontaneous splenic rupture must be suspected in the emergency room who present with abdominal pain and hemodynamic instability and who have used especially anticoagulants and anti-platelet medications without a recent history of trauma. One of the important causes of mortality is a missing or delayed diagnosis [7,9].

After considering factors related to the index bleeding event, the underlying thromboembolic risk, and comorbid conditions, a decision to accept or modify the anticoagulation should be made in a multidisciplinary approach, along with other care team members, the patient, and their caregivers. For gastrointestinal bleeding, some evidence suggests 14 days to resume anticoagulation to prevent recurrent bleeding, thromboembolism, and mortality. In the case of intracranial hemorrhage, resumption is within a month; however, in the case of splenic rupture, there is no specific consensus, and there should be a clinical decision-making process personalized to each patient [20].

## Conclusions

The mechanisms by which spontaneous splenic rupture occurs in patients with anticoagulation with direct oral anticoagulants are unknown. It may be difficult to recognize a possible splenic rupture, especially if patients don’t have risk factors like prior trauma or infectious or lymphoproliferative disease. 

It is important to suspect splenic rupture in patients with abdominal pain associated with hemodynamic instability who are receiving anticoagulation so that management can be done promptly. More research is needed to determine guidelines on the treatment and prevention of this complication, as well as the importance of developing retrospective or prospective studies to determine the risk of recurrence in patients with a history of splenic rupture or other major bleeding. There is no clear consensus on whether to restart anticoagulation after this type of event. Since much of the research is done for gastrointestinal or intracranial hemorrhage, more research is warranted regarding splenic rupture.
